# The role of glyoxalases for sugar stress and aging, with relevance for dyskinesia, anxiety, dementia and Parkinson's disease

**DOI:** 10.18632/aging.100258

**Published:** 2011-01-16

**Authors:** Georg Auburger, Alexander Kurz

**Affiliations:** Exp. Neurology, Department of Neurology, Goethe University Medical School, 60590 Frankfurt am Main, Germany

Carbohydrates are the primordial source of energy and carbon for certain unicellular organisms as well as for the complex mammalian brain and its synaptic functions [1]. However, for all these cells the degradation of carbohydrates poses several problems, e.g. the formation of toxic by-products such as the glycating electrophile methylglyoxal (MG, also named 2-oxo-propanal) or the excessive generation of lactic acid with ensuing pH change [2]. Under conditions of high carbon flux and under limited availability of NAD+, e.g. during anaerobic glycolysis [3], the triose phosphates dihydroxyacetone phosphate and glyceraldehyde-3-phosphate spontaneously decompose to MG, a compound known to contribute to the generation of advanced glycation endproducts (AGEs) [4] – with possibly irreversible damage to lipids, nucleic acids and proteins, in particular of mitochondria [5] – and to the induction of ubiquitin conjugates [6]. In a pathway with very high conservation during evolution, all these cells use the enzyme glyoxalase I (lactoylglutathione lyase) in the presence of glutathione (GSH) to convert MG into S-lactoyl-glutathione (SLG) and then use glyoxalase II (hydroxyacylglutathione hydrolase) to liberate D-lactate and glutathione. In spite of this toxicity of MG, some bacteria use the enzyme methylglyoxal synthase to generate MG, apparently to regulate carbon flux and growth rate [7]. Furthermore, the ratio between GSH and SLG in such bacteria modulates potassium efflux and intracellular acidification [8]. Acting as a signal initiator, MG activates the osmosensor Sln1, the HOG-MAP kinase cascade and the calcium(2+) signalling pathway in yeast [9]. In human cells, MG has a well established anti-proliferative effect in cancerous cells with enhanced glycolysis (Warburg-effect) [10, 11]. MG was recently reported to play a physiological role in the modulation of hypoxia-induced-factor-1α (HIF-1alpha) levels and thus in the modulation of the balance between anaerobic and aerobic bioenergetics [12]. Interestingly, an anxiety-suppressing effect of MG infusions into the brain was observed in mice [13]. And in patients with ditary dyskinesias, episodic disorders of spontaneous movement, it is now known that either defects in the cerebral glucose transport or defects in the putative neuronal SLG sensor protein MR-1 can be responsible for these symptoms [14,15]. These observations suggest that MG and SLG are not simply toxic by-products to be eliminated, but might play an important physiological function in bioenergetic signaling.

In view of the importance of bioenergetics, sugar stress and growth pathways for the molecular mechanisms of aging, it is hardly surprising that mutations in the glyoxalase pathway affect the survival under carbonyl stress and adverse growth conditions. A stress-protection effect of glyoxalase I overexpression or a sensitizing effect of glyoxalase I deficiency was alredy documented in the bacteria Escherichia coli under anaerobic conditions with high carbon flux [16], the protozoan parasite *Leishmania donovani* [17], in the yeast *Saccharomyces cerevisiae* [18], in the plant *Nicotiana tabacum* [19,20], in the worm *Caenorhabditis elegans* [21] and in *Rattus norvegicus* and *Homo sapiens* under diabetic sugar stress [22,23].

The attention of investigators was focused on glyoxalase I, since this enzyme catalyzes the rate-limiting step in the pathway. Furthermore, glyoxalase I is regulated in its expression on the transcript level. Elevated mRNA / protein levels or increased enzyme activity were found to mediate the survival of dental caries bacteria *Streptococcus mutans* and their continued maintenance of glycolysis in media with high sugar and acid concentrations [24,25], of *Bacillusanthracis*, of the fungus *Candida albicans* and of the worm *Onchocerca volvulus* under oxidative stress [26-28], of *Arabidopsis thaliana* under abiotic stress [29], of the high-dormancy seeds of the grass*Lolium rigidum* Gaud [30], of rice *Oryza sativa* roots under chilling temperatures or leaves under UV radiation [31,32], of the tomato *Solanum pimpinellifolium* as well as the mustard *Brassica juncea* under salt and heavy metal stress [33,34] and of mammalian cancer cells under stress [35]. However, it is has remained unclear whether increased glyoxalase I levels only serve to protect normal growth and survival under adverse conditions, or whether they are able to postpone aging and increase health at advanced age, as a means to longevity.

Therefore, the recent publication in AGING from investigators around H. Osiewacz in Frankfurt [36] is therefore very important, assessing the relevance of the glyoxalase pathway for growth and lifespan in a model organism that has been very well characterized for more than 50 years within the field of healthy aging organism research, namely the fungus *Podospora anserina* [37]. This filamentous ascomycete thrives under aerobic conditions and on extracellular glucose, completing a life cycle of germination, maturity with maximal growth, senescence with pigmentation and apical cell death within three weeks. Here, the principal finding of the authors was the extension of the healthy lifespan by 4.3% through the combined overexpression of glyoxalase I + II under conditions of sugar stress (2% glucose), while no lifespan differences were apparent for normal sugar concentrations. No growth differences were apparent under any conditions tested. Importantly and unexpectedly, this study found a reduction of lifespan through strong overexpression of glyoxalase I alone, in contrast to a report of enhanced lifespan after mild glyoxalase I overexpression in *C. elegans* [21], suggesting that overly strong glyoxalase activity may be toxic due to glutathione depletion with subsequent oxidative stress and possibly potassium efflux. Together with three crucial previous publications from the same team [38-42], these data indicate that a prolongation of healthspan is possible through a network of factors controlling aerobic and anaerobic bioenergetics.

Indeed, these data are of paramount importance for the needs of our modern civilization characterized by excess sugar consumption and an ever older population. They are also a sound scientific basis for future molecular understanding of human aging, where the glyoxalase system has been implicated in senescence and in several age-related disorders. In man, where the promoter of gloxalase I is inducible by insulin and heavy metals [43], the transcript levels of glyoxalase I show a continuous decrease after the start of senescence at 55 years of age, similar to the decrease of glyoxalase I levels in old rat muscle tissue [44,45]. As a biomarker of anxiety, altered expression levels of glyoxalase I were reported in mice, compatible with the hypothesis that low MG levels correlate with anxiety, and an anxiolytic effect of MG infusions into brain was demonstrated [13,46-49]. Glyoxalase I anomalies were also reported in psychiatric diseases such as mood disorder, schizophrenia and autism, where anxiety symptoms are altered [50-52]. As a biomarker of tau protein pathology in Alzheimer's disease and frontotemporal dementia, increased expression of glyoxalase I was reported in mouse models and patients, and tau aggregation was observed as an effect of elevated MG levels [53-55]. With tau and alpha-synuclein being the two most important genetic risk factors of Parkinson's disease (PD) [56] and in view of the co-precipitation of these two proteins in the pathognomonic cytoplasmic “Lewy body” inclusions [57,58], it is interesting that we found an upregulation of glyoxalase I to be the prominent response within the mouse brain transcriptome to a deficient alpha-synuclein function [59]. Whether a gain-of-function of alpha-synuclein and other Parkinson triggering events also modulate the glyoxalase system, remains an issue we are investigating, and this seems credible in view of a report on altered glyoxalase I expression in mouse brains with Parkin deficiency [60]. Furthermore, an unexplained glutathione depletion and the presence of AGEs in brain autopsies with incidental Lewy bodies [61,62] suggests an involvement of this pathway in the earliest stages of PD pathogenesis.

Since pharmacological tools are available to selectively antagonize glyoxalase I function [10,11,63] and to relieve carbonyl stress [64-67], it is now very promising to have the mutants generated in this publication of AGING [36] as a fast and well-characterized micro-organism model of age-related neurodegenerative disease, and to use them to understand and postpone this pathological process.
